# Ivermectin-treated cattle reduces blood digestion, egg production and survival of a free-living population of *Anopheles arabiensis* under semi-field condition in south-eastern Tanzania

**DOI:** 10.1186/s12936-017-1885-x

**Published:** 2017-06-06

**Authors:** Issa N. Lyimo, Stella T. Kessy, Kasian F. Mbina, Ally A. Daraja, Ladslaus L. Mnyone

**Affiliations:** 1 Environmental Health and Ecological Sciences Department, Ifakara Health Institute, Off Mlabani Passage, P.O.BOX 53, Ifakara, Morogoro United Republic of Tanzania; 2 Pest Management Centre, Sokoine University of Agriculture, Morogoro, Tanzania; 3 School of Public Health, University of the Witwatersrand, Parktown, Republic of South Africa

**Keywords:** Ivermectin, Cattle, *Anopheles arabiensis*, Exophagy, Zoophagy, Blood-digestion, Haematin, Egg-production, Survival, Residual-transmission, Vector–control

## Abstract

**Background:**

*Anopheles arabiensis* feed on cattle and contributes to residual transmission of malaria in areas with high coverage of long-lasting insecticide-treated nets and indoor residual spraying in East Africa. This study aimed to evaluate the effects of ivermectin-treated cattle as a complementary vector control tool against population of *An. arabiensis* under the semi-field conditions in south-eastern Tanzania.

**Methods:**

The free-living population of *An. arabiensis* was allowed to forage on untreated or ivermectin-treated cattle in alternating nights within the semi-field system in south-eastern Tanzania. Fresh blood fed mosquitoes were collected in the morning using mouth aspirators and assessed for their blood meal digestion, egg production, and survivorship. The residual activity of ivermectin-treated cattle was also determined by exposing mosquitoes to the same treatments after every 2 days until day 21 post-treatments. These experiments were replicated 3 times using different individual cattle.

**Results:**

Overall, the ivermectin-treated cattle reduced blood meal digestion in the stomach of *An. arabiensis*, and their subsequent egg production and survival over time. The ivermectin-treated cattle halved blood meal digestion in mosquitoes, but reduced their egg production for up to 15 days. The ivermectin-treated cattle reduced the survival, and median survival times (1–3 days) of *An. arabiensis* than control cattle. The daily mortality rates of mosquitoes fed on ivermectin-treated cattle increased by five-fold relative to controls in the first week, and it gradually declined up to 21 days after treatment.

**Conclusion:**

This study demonstrates that long-lasting effects of ivermectin-treated cattle on egg production and survival of *An. arabiensis* may sustainably suppress their vector density, and reduce residual transmission of malaria. This study suggests that ivermectin-treated non-lactating cattle (i.e. calves, heifers and bulls) could be suitable option for large-scale malaria vector control without limiting consumption of milk and meat by communities in rural settings. Furthermore, simulation models are underway to predict the impact of ivermectin-treated cattle alone, or in combination with LLIN/IRS, the frequency of treatment, and their coverage required to significantly suppress population of *An. arabiensis* and reduce residual transmission of malaria.

## Background

Malaria vector control tools such as the long-lasting insecticide-treated nets (LLIN) and indoor residual spraying (IRS) have controlled malaria transmission in Africa by targeting mosquitoes that blood feed on humans (anthrophagy, anthrophily), and rest inside houses (endophagy, endophily) [1, 2]. Specifically, these indoor interventions reduced feeding frequency, density and survival of *Anopheles gambiae* s.s. [3–7] and *Anopheles funestus* [6, 8, 9]. However, transmission risk and burden of malaria in Africa is yet unacceptably high even in areas with high coverage of LLIN and IRS [10–14], because of physiological insecticide resistance [11, 14, 15], and behaviours of outdoor feeding (exophagy), resting (exophily); biting in the early evening and morning, feeding on animals and resting in cattle shelters (zoophagy, zoophily) in Anopheles mosquitoes [6, 7, 11, 12, 14, 16–19]. Therefore, a novel vector-control strategy capable of reducing the density and survival of outdoor biting, and zoophilic mosquitoes is urgently required to complement LLIN and IRS by controlling residual transmission of malaria.

Endectocides, such as ivermectin, which are broad-spectrum systemic drugs against nematodes and arthropods in public [20–23], and veterinary health importance [24], they are also potential novel vector-control tools for targeting outdoor biting, and insecticide resistant *Anopheles* mosquitoes [25–27]. However, the effects of ivermectin-treated humans against malaria vectors have been extensively investigated relative to treated-cattle. Ivermectin act on glutamate gated chloride channels in parasites including mosquitoes [28–32], which differ from target sites of conventional insecticides used in LLIN and IRS. Therefore, mass drug administration (MDA) of ivermectin to humans may targets malaria vectors regardless of their biting locations [16, 17, 33], time [17, 19, 34], and physiological insecticide resistance status [11, 14, 15]. Laboratory studies demonstrated that ivermectin-treated human blood reduces feeding frequency, blood meal digestion, rate of defecation, survival, fecundity, vector density, and sporozoite rates of mosquitoes after membrane or direct feeding assays [35–40]. Similarly, the MDA of ivermectin to humans may decrease malaria transmission by reducing the survival of wild Anopheles mosquitoes for 1 week that lead to increased young females for 3 weeks, and reduced sporozoite rates for 2 weeks [41–45]. Even if long-lasting formulations of ivermectin administered to humans could sustain reduction in malaria transmission, it may be inefficient to suppress malaria vectors that blood feed on cattle.

Zoophilic vectors contribute to malaria transmission in many parts of the world such as *Anopheles arabiensis* in Africa [46], *An. albimanus* in Latin America [47], *An. sinensis* in Asian-Pacific [48]. For example, *An. arabiensis* feed on cattle and continues to transmit malaria outside houses in areas with high coverage of LLIN and IRS across East-Africa [6, 7, 49–51]. Treating cattle with ivermectin could control these mosquitoes [52–54], yet the studies that evaluated this strategy in more realistic environments are scarce. For example, few laboratory and field studies demonstrated that ivermectin-treated cattle reduced the survival and fecundity of Anopheles mosquitoes (e.g. *An. gambiae* s.s, *An. arabiensis*, *Anopheles coluzzii*, *Anopheles culicifacies* and *Anopheles stephensi*) [55–59]. Although the effects of subcutaneous-treated cattle to mosquitoes were long-lasting than orally- or topically-treated cattle [55, 57–59], most of these studies used laboratory mosquitoes and artificial feeding strategies which have excluded mosquito genetic and phenotypic diversity [60–62], and vertebrate host ecology [63]. The blood feeding behaviour of *An. arabiensis* on cattle has genetic basis [51, 61], but ivermectin-treated cattle against these mosquitoes has never been evaluated using host-mosquito interactions as in the natural environments. Therefore, several knowledge gaps including the effects of ivermectin-treated cattle against fitness of *An. arabiensis* remain to be studied under semi-field conditions before recommending for a large-scale trial.

We evaluated the effects of ivermectin-treated cattle against free-flying population of *An. arabiensis* within the semi-field system that is closely related to the natural environments in south-eastern Tanzania. The specific objectives were: (1) to demonstrate that ivermectin-treated cattle reduce blood meal digestion and the subsequent egg production, and survival in *An. arabiensis*, and (2) to assess the duration of these effects (residual activity) against these mosquitoes. This information will be useful in assessing the appropriateness of ivermectin-treated cattle as complementary to LLIN and IRS for controlling outdoor-biting, and insecticide-resistant *An. arabiensis*.

## Methods

### Study site

The study was conducted within the semi-field systems (SFS) at the Ifakara Health Institute (IHI) in the Kilombero Valley, south-eastern Tanzania [64]. Although three major African malaria vectors (*An. gambiae* s.s, *An. funestus* and *An. arabiensis*) are found in this valley [65–69], *An. arabiensis* that composes >90% of the population in most of the villages contributes to outdoor transmission of malaria [3, 51]. Throughout this valley, majority of cattle shelters are kept at 2.5–30 m from human dwelling houses (Lyimo et al., unpublished data). The previous analysis of blood fed mosquitoes revealed that 80% of *An. arabiensis* obtain blood from cattle and rest outside houses [51], but this exophily and zoophagy has genetic basis [61].

### Mosquitoes

Experiments were conducted using a self-sustaining population of *An. arabiensis* surviving within a large SFS (21 × 9.1 × 7.1 m) at the IHI since 2008 [62, 70]. This population was established from individuals of wild mosquitoes collected from a nearby Sagamaganga village (~15 km from IHI). Within this system, mosquitoes have access to a range of natural habitat including cattle blood sources, larval habitat, vegetation and animal shelters. These mosquitoes express similar patterns of larval development, mating, feeding and resting behaviour [62]; genetic and phenotypic diversity; and reduced inbreeding as the wild population in the field [60, 62].

### Cattle and treatments

Livestock owners from nearby local communities surrounding IHI compound provided their cattle for these experiments. Cattle were collected after the purpose of the experiments was explained to livestock owners, and those accepted for their animals to participate in the trial were required to fill the written informed consent. Majority of cattle (>88%) in Kilombero Valley are sprayed with irritant, repellent pyrethroids, especially Alphacypermethrin (Lyimo et al., unpublished data). Therefore, cattle that had no history of being sprayed with any insecticide for the past 2–3 months were chosen for these experiments. To avoid residues of pyrethroids on cattle, all cattle were transferred to the IHI compound, and washed with water for 3 days consecutively prior to the start of experiments.

A total of 6 cattle were divided into two groups of 3 individuals: untreated (control) and ivermectin-treated group. The weight of individuals in treated group was estimated by veterinarians using girth tape, and then subcutaneously injected with commercially available 1% ivermectin solution (IVOMEC^®^) at a therapeutic dose of 0.2 mg/kg body weight. Another group of three individuals remained untreated as control during these experiments.

### Experimental procedures

#### Evaluating the effects of ivermectin-treated cattle

**Fig. 1:**
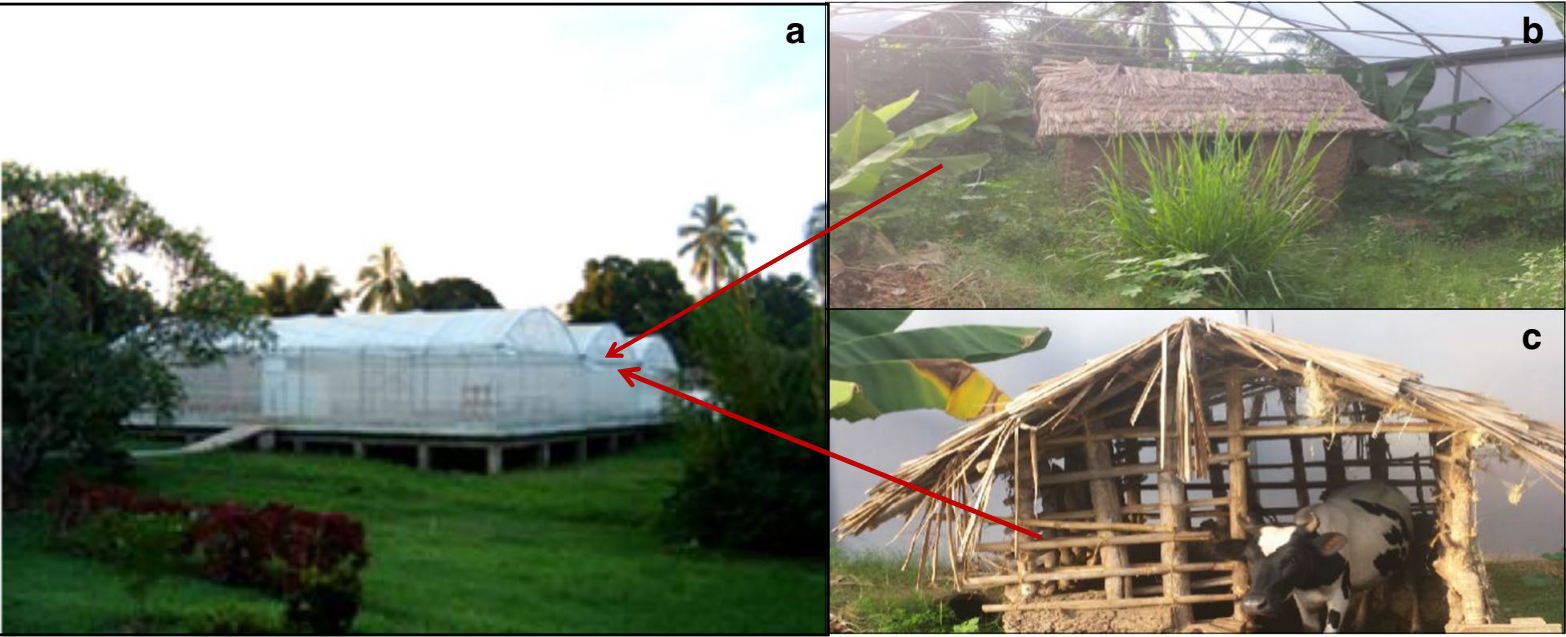
Picture of a semi-field system showing: **a** the* outside view* of the chamber where free living population of *Anopheles arabiensis* was established, **b** the* inside* of the experimental chamber with established vegetations, breeding and resting habitats for mosquitoes, and **c** the shelter with cattle as the source of blood meal for *An. arabiensis*

A population of *An. arabiensis* within the chamber of the SFS was exposed to forage on untreated or ivermectin-treated cattle from 5:30 p.m. to 5:30 a.m. in alternating nights (Fig. 1). Every morning, all fresh blood fed mosquitoes were collected using torches and mouth aspirators from inside cattle shelters, and clay pots. These freshly blood fed mosquitoes were distinguished from those fed the previous night on another treatment by visual observations of the blood meal digestion in the stomach and egg development that requires 2–4 days [71–73]. The blood colour in the mosquito abdomen changes with time: full reddish to dark red abdomen after 6 h (fresh fed), black three-quarter abdomen after 12 h, black two-third to anterior half of abdomen after 24–36 h (semi-gravid), and black in ventral side of abdomen to no blood after 48–72 h (gravid) [74, 75]. All semi-gravid and gravid mosquitoes, if captured, were considered to have fed the previous nights on another treatment, and thus they were excluded from sample of fresh blood fed mosquitoes. Mosquito development time between generations under this SFS was previously estimated to range from 21 to 25 days [62]. Therefore, the experimental replicates/blocks were spaced by 3 weeks to ensure adequate sample sizes of mosquitoes. These experiments were replicated three times using different cattle individuals (2 individual cattle/replicate × 3 replicate = 6 individual cattle). The impact of ivermectin-treated cattle on blood meal digestion and subsequent egg production, and survival of *An. arabiensis* was quantified as follows:

##### Blood meal digestion

The digestion of blood proteins (haemoglobin) concentrated in the abdomen of Anopheles mosquitoes produces large amount of potentially toxic molecule (heme) that is detoxified and defecated as haematin crystals [76–79]. The estimate of mass of haematin defecated by mosquitoes at the end of digestion is positively correlated with the amount of blood proteins ingested. Thus, the effects of ivermectin on the efficiency of blood meal digestion in the mosquito stomach reflected by the mass of haematin defecated was quantified following methodology of Briegel [80]. In this method, blood fed mosquitoes were individually transferred into 30 ml universal tubes that were covered with netting at the top (haematin tubes), and they were provided with a 10% glucose solution using a strip of damp cotton wool placed on top of the tube. These mosquitoes were left for 4 days in haematin tubes because they require 2–4 days to complete digestion of blood meal and egg development [71, 72]. On the fourth day, the haematin sample deposited by mosquitoes at the bottom of 30 ml universal tubes was dissolved by 1% lithium carbonate to quantify the amount of haematin defecated by mosquitoes [80].

##### Egg production

After collection of haematin samples, mosquitoes were transferred into paper cups with wet filter paper at the bottom for laying eggs. These mosquitoes in the cups were provided with 10% as in haematin tubes, and checked daily to identify if and when they laid eggs. The mosquitoes that laid eggs were counted to determine their oviposition rates (proportion laid eggs). The eggs were also were also counted under dissecting microscope at ocular magnification of 10× to determine mosquito fecundity (number of eggs produced by mosquitoes).

##### Survivorship

The individual mosquitoes were monitored for their daily survival after blood feeding while in the haematin tubes, and fecundity cups. All mosquitoes that laid eggs were transferred into dry paper cups (survival cups), and continued to be monitored to determine their day of death after blood feeding. The numbers of days survived by mosquitoes were recorded.

#### Assessing the residual activity of ivermectin-treated cattle

The above experiments were repeated to assess the duration of effects (residual activity) of ivermectin by exposing same individuals of untreated, or ivermectin—treated cattle to the population of *An. arabiensis* at different time points such as 3, 6, 9, 12, 15, 18 and 21 post-treatment days. The treatments were exposed to mosquitoes in alternating days within 3-week long experimental block/replicate as described in experiment 1 above. For each repetition, freshly bloods fed mosquitoes were sampled and assessed their blood meal digestion, egg production, and survival as described in experiment 1 above. These experiments replicate were replicated three time using different individuals of cattle (2 cow individuals/replicates × 3 replicates = 6 cow individuals) as in the experiment 1 above.

### Statistical analyses

Statistical analysis was conducted to demonstrate the effects of ivermectin-treated cattle on the fitness of *An. arabiensis*, and assess the residual activity of treatments using natural host-mosquito interactions within the semi-field system. Four key parameters were analysed: efficiency of blood meal digestion (mass of haematin defecated), proportion of mosquitoes laid eggs (oviposition rate), number of eggs (fecundity), and number of days survived by mosquitoes (post-exposure survival of mosquitoes).

The effects of ivermectin-treated cattle, the post-treatment time, and their interaction on the continuous (i.e. mass of haematin defecated, and number of eggs), and binomial response variable (proportion laid eggs) of female *An. arabiensis* were respectively analysed by fitting generalized linear mixed effect model (glmer) with Poisson (log link) and binomial (logit link) errors in the lme4 statistical package in R version 3.1.1 [81]. Firstly, the effects of ivermectin-treated cattle on the response variables were assessed by using model with cattle ‘individuals’, and ‘experimental night’ as random effects, and ‘Treatment’ as the main effects. The null model built with ‘random effects’ was compared with full model composed of both ‘random effect’ and ‘main effects’ using ‘ANOVA’ to identify statistical significant effects of ‘Treatment’. Lastly, the effects of ivermectin-treated cattle ‘Treatment’ on the response variables across time were analysed by sequential addition of the main effects including ‘Treatment’, ‘post-treatment time’ and their interaction into a null model (forward selection). Then likelihood ratio test (LRT) was used to identify if the addition of main effects into a null model lead into a statistical significant improvement of the model. If the interaction between ‘treatment’ and ‘post-treatment time’ was significant, then the main effects of treatment was analysed for each post-treatment time to establish the time point with statistical significant impact on response variables.

The continuous response variable of survival of mosquitoes (number of days survived) after blood feeding on untreated or ivermectin-treated cattle was analysed using survival package in the statistical software of R version 3.1.1 [81]. The Cox Proportional Hazards Model (coxph) in the survival package compares between survival curves using Hazard Ratio (HR). In this model, a frailty function was used to incorporate the random effect of ‘individual cows’ or ‘replicate’ to form null model. Therefore, the main effect of ‘Treatment’ was added into a null model, and tested which random effect has statistical significant improvement of the model. The full model was used to estimate hazard ratio (risk of death) to identify statistical significant differences between Kaplan–Meier survival curves. These survival curves were used to generate median survival times of mosquitoes after blood feeding on ivermectin-treated or untreated cattle. Further analysis of survival data was conducted to investigate how the impact of ivermectin on the survival of mosquitoes changes over time. The Cox Proportional Hazard Model (coxph) was composed of frailty function to incorporate the random effect of ‘individual cows’ or ‘block’, ‘treatment, ‘post-treatment time’, and their interactions (treatment*post-treatment time) were fit as main effects in R statistical software. These terms were sequentially added into the null model, and tested if there is statistical significant improvement. When the interaction term of ‘treatment*post-treatment time’ was statistically significant, the Cox Proportion Hazard Models (coxph) were fit with main effect of treatment and random effects of ‘individual’ for each post-treatment time to identify statistical significant differences between survival curves of mosquitoes after blood feeding on untreated or ivermectin-treated cattle. Then the Kaplan–Meier survival function was used to generate survival curves, and to estimate median survival times for each post-treatment time.

## Results

A total of 1136 freshly blood fed mosquitoes were collected from self-sustaining population of *An. arabiensis* after exposure to untreated, and ivermectin-treated cattle (Table 1). The effects of ivermectin-treated cattle on mosquito blood meal digestion and their subsequent egg production and survival were presented as follows:

**Table 1:** The sample size of blood fed *An. arabiensis* collected from the population within the semi-field system

Number of blood fed	*An. arabiensis* collected from the semi-field after exposure to untreated or ivermectin treated cattle		
Replicate	Days post-treatment	Control	Treatment
1	0	44	13
1	3	17	5
1	6	11	13
1	9	21	22
1	12	23	17
1	15	23	28
1	18	24	18
1	21	18	28
2	0	35	56
2	3	36	32
2	6	24	32
2	9	17	39
2	12	10	18
2	15	20	28
2	18	18	32
2	21	23	36
3	0	25	19
3	3	30	26
3	6	15	14
3	9	22	20
3	12	12	20
3	15	21	21
3	18	36	33
3	21	24	17
Total		*549*	*587*

### Effects on blood meal digestion

**Fig. 2:**
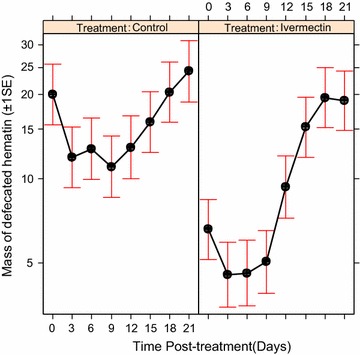
Estimated mass of haematin defecated (±1 s.e) by *An. arabiensis* population after feeding on control and ivermectin-treated cattle across time. The relationship between the effects of ivermectin treated cattle and post-treatments time on mass of haematin defecated by mosquitoes at the end of blood meal digestion

Blood meal digestion as estimated by the mass of haematin defecated by *An. arabiensis* was significantly influenced by the treatment of cattle with ivermectin ($\upchi_{1}^{2}$ = 27.26, P < 0.01, Fig. 2), post-treatment time ($\upchi_{6}^{2}$ = 2077.16, P < 0.01, Fig. 2), and their interactions (ivermectin treatment*post-treatment time: ($\upchi_{7}^{2}$ = 594.45, P < 0.001, Fig. 2). The efficiency of blood meal digestion in mosquitoes fed on ivermectin-treated cattle was significantly reduced by half compared to the mosquitoes fed on control cattle at 6 days post-treatments ($\upchi_{1}^{2}$ = 4.06, P = 0.04, Fig. 2), and the other post-treatment time shown a non-significant decreasing trend (P > 0.05, in all cases, Fig. 2).

### Effects on egg production

**Fig. 3:**
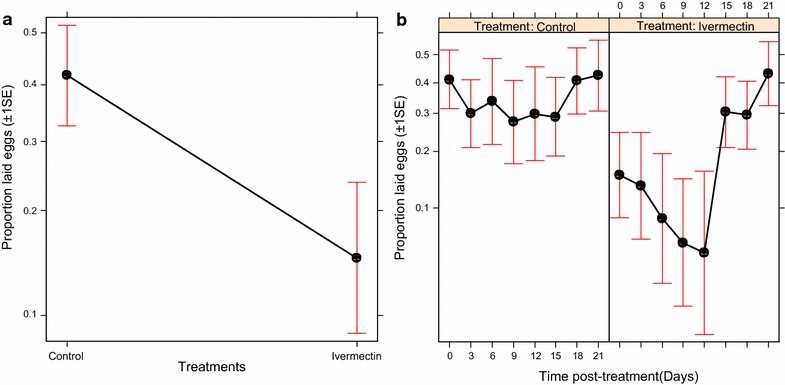
Estimated proportion (±1 s.e) of *An. arabiensis* laid eggs after blood feeding on control and untreated cattle: **a** the effects of ivermectin-treated cattle on proportion of mosquitoes laid eggs, and **b** the relationship between the effects of ivermectin-treated cattle and post-treatment time on proportion of mosquitoes laid eggs

The probability that *An. arabiensis* laid eggs after blood feeding was significantly affected by ivermectin-treated cattle ($\upchi_{1}^{2}$ = 9.35, P < 0.001, Fig. 3a), post-treatment time ($\upchi_{6}^{2}$ = 30.82, P < 0.001, Fig. 3b), and their interactions (treatment*post-treatment time: $\upchi_{8}^{2}$ = 36.99, P < 0.001, Fig. 3b). The oviposition rates of *An. arabiensis* were significantly reduced by 64.61% after blood feeding on ivermectin-treated cattle relative to those fed on control cattle, but it changed across time (Fig. 3a, b). The oviposition rates of *An. arabiensis* fed on ivermectin-treated cattle was reduced relative to those mosquitoes fed on control cattle by 54.64% at day 3 ($\upchi_{1}^{2}$ = 5.58, P = 0.02), 74.14% at day 6 ($\upchi_{1}^{2}$ = 5.96, P = 0.01), 76.87% at day 9 ($\upchi_{1}^{2}$ = 5.49, P = 0.02) and 81.62% at day 12 ($\upchi_{1}^{2}$ = 6.95, P < 0.01) post-treatments (Fig. 3b), but the effects gradually decreased up until 15 days post treatment (P > 0.05, Fig. 3b).

**Fig. 4:**
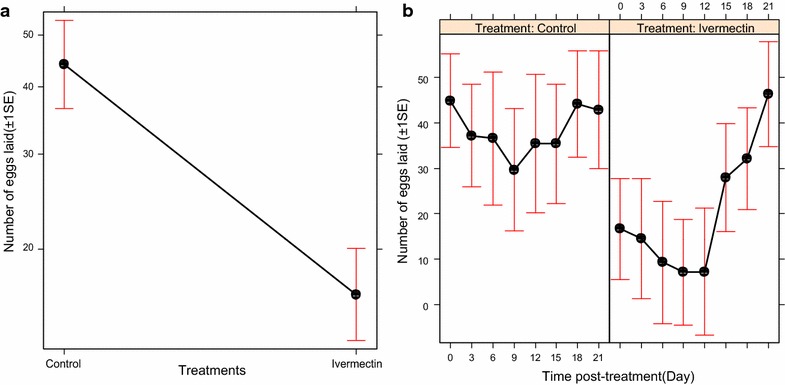
Estimates (±1 s.e) of the mean number of eggs laid by *An. arabiensis* after obtaining blood meal from control and ivermectin-treated cattle: **a** effects of ivermectin-treated cattle on fecundity of mosquitoes, and **b** the relationship between the effects of ivermectin-treated cattle and post-treatment time on fecundity of mosquitoes

Similarly, the fecundity of *An. arabiensis* was significantly influenced by the treatment of cattle with ivermectin ($\upchi_{1}^{2}$ = 13.25, P < 0.001, Fig. 4a), post-treatment time ($\upchi_{6}^{2}$ = 18.28, P < 0.01, Fig. 4b), and their interactions (treatment*post-treatment time: $\upchi_{8}^{2}$ = 23.60, P < 0.01, Fig. 4b). The ivermectin-treated cattle reduced fecundity of mosquitoes by 62.98% relative to mosquitoes that fed on control cattle (Fig. 4a). Over time, the ivermectin-treated cattle reduced fecundity of *An. arabiensis* relative to control cattle by 60.89% at day 3 ($\upchi_{1}^{2}$ = 5.88, P = 0.01), 74.71% at day 6 ($\upchi_{1}^{2}$ = 6.75, P < 0.01), 75.20 at day 9 ($\upchi_{1}^{2}$ = 6.20, P = 0.01) and 79.67% at day 12 ($\upchi_{1}^{2}$ = 6.98, P < 0.01) post treatments, but the reduction declined slowly up until 15 days post-treatments (P > 0.05, in each case, Fig. 4b).

### Effects on longer-term survival

**Fig. 5:**
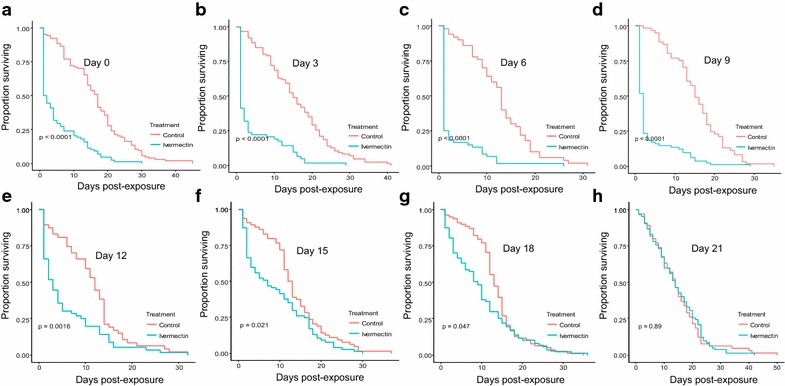
Estimates of the survival of *An. arabiensis* after blood feeding on control and ivermectin-treated cattle. The effects of ivermectin-treated cattle on survival of mosquitoes were estimated at different time points: **a** Day zero, **b** Day 3, **c** Day 6, **d** Day 9, **e** Day 12, **f** Day 15, **g** Day 18, and **h** Day 21 post-treatment. The *lines* represent the survival function as estimated from the fitting Cox proportional hazard model (controlling for random variation of between individual cattle)

**Fig. 6:**
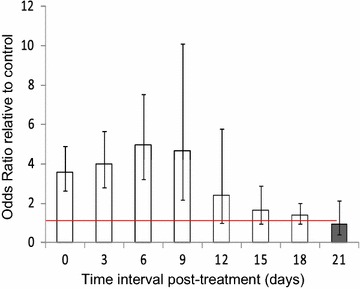
Estimates of odds of mortality of *An. arabiensis* after blood feeding on control and ivermectin-treated cattle

The long-term survival of *An. arabiensis* was significantly influenced by the ivermectin-treated cattle ($\upchi_{1}^{2}$ = 142.54, P < 0.001, Fig. 5), post-treatment time ($\upchi_{7}^{2}$ = 101, P < 0.001, Fig. 5), and their interactions (treatment*post-treatment time: ($\upchi_{8}^{2}$ = 87.67, P < 0.001, Fig. 5). The long-term survival of *An. arabiensis* was significantly reduced by 52.53% after blood feeding on ivermectin-treated relative to mosquitoes that fed on control cattle ($\upchi_{1}^{2}$ = 62.87, P < 0.001, Fig. 5a). The proportion of surviving *An. arabiensis* and their median survival times after blood feeding on ivermectin-treated cattle was significantly reduced relative to mosquitoes that fed on control cattle (Fig. 5a–c; Tables 2, 3), but they remained lower than on control cattle until at day 21 post-treatments (Fig. 5d–h; Tables 2, 3). Furthermore, the daily mortality rates of mosquitoes that fed on ivermectin-treated cattle increased from 50 to 80% relative to mosquitoes that fed on control cattle, and then slowly declined up until 21 days post-treatment. The ivermectin-treated cattle increased risk of death of mosquitoes by five-folds relative to control cattle (P < 001 in most cases, Tables 2, 3; Figs. 5, 6), but such risk gradually decreases with time up until 21 days (P > 0.05, Tables 2, 3; Figs. 5, 6).

**Table 2:** Mortalities of *An. arabiensis* after blood feeding on control and ivermectin treated cattle

Mortality rates (%) of *An. arabiensis* after blood feeding on control or ivermectin treated cattle		
Days post-treatment	Control cattle	Treated cattle
0	4.81	50
3	3.49	58.73
6	5.71	80.43
9	2.56	59.02
12	3.49	33.33
15	4.81	10.71
18	2.38	11.11
21	2.28	11.11

**Table 3:** Estimated median survival times of *An. arabiensis* after blood feeding on control and ivermectin-treated cattle within the semi-field systems

Median survival of *An. arabiensis* after blood feeding on control or ivermectin treated cattle		
Days post-treatment	Control cattle	Treated cattle
0	17 (14–18)	1.5 (1–4)
3	15 (13–19)	1 (1–2)
6	13 (11–15)	1 (1–1)
9	15 (13–18)	2 (1–2)
12	12 (10–14)	3 (2–5)
15	12.5 (11–15)	7 (3–12)
18	13 (12–15)	9 (6–10)
21	14 (11–17)	14 (12–17)

Numbers in brackets are 95% confidence intervals of median survival

## Discussion

This study demonstrates that the ivermectin-treated cattle reduce blood meal digestion, and subsequent egg production, and survival of free-living population of *An. arabiensis* in South-Eastern Tanzania. The ivermectin-treated cattle decreased the efficiency of blood meal digestion in *An. arabiensis*, and their subsequent egg production for up to 2 weeks. The proportion surviving and their median survival times of these mosquitoes were reduced after blood feeding on ivermectin-treated cattle than control cattle for up to 3 weeks. Additionally, ivermectin-treated cattle increased daily mortality rates of *An. arabiensis* by five folds than control cattle, but it gradually declined for up to 3 weeks post-treatment. These results imply that the effects of ivermectin-treated cattle on efficiency of blood meal digestion, egg production, and survival of population of *An. arabiensis* may suppress their vector density and reduce outdoor transmission of malaria.

The digestion of blood proteins (haemoglobin) in the mosquito stomach generates two key products: heme that is detoxified and defecated as haematin, and essential nutrients for egg production [77–79]. The present study found that ivermectin-treated cattle reduced both oviposition rates and fecundity of *An. arabiensis* than untreated cattle for 15 days post-treatments. This reduction in mosquito egg production may be explained by the decreased mass of haematin defecated by these mosquitoes at the end of digestion suggesting that small amount of blood proteins in their stomach was converted to less nutrients required for egg development. Perhaps ivermectin changes digestive responses to blood meal in mosquito stomach (e.g. malformation of peritrophic matrix) that lead to reduced efficiency of blood digestion, defecated haematin, and nutrients for egg production. For instance, previous laboratory studies confirmed that ivermectin, chitinase, and silencing disrupted peritrophic matrix in blood fed mosquitoes or sand flies that reduced their blood meal digestion, haematin defecation, and their subsequent egg production [36, 82–84]. Additionally, our finding is consistent with previous studies that observed ivermectin-treated cattle decreased egg production in *An. coluzzii* [57], *An. gambiae* s.s [55], and *An. arabiensis* [55, 56], for up to 10 days after subcutaneous injection [55, 57]. The present study suggests that ivermectin-treated cattle may reduce digestion of blood proteins in mosquito stomach resulting to small amount of defecated haematin, and essential nutrients absorbed for egg production.

This study also demonstrated that the negative effects of ivermectin-treated cattle on the long-term survival of *An. arabiensis* was strong, and declined with post-treatment time. The ivermectin-treated cattle reduced the proportion of surviving *An. arabiensis*, and their median survival times for up to 3 weeks after treatment. Similarly, the ivermectin treated cattle increased daily mortality rates by five-folds than control cattle, but the risk of death gradually declined until 3 weeks post-treatments. The possible explanation could be that ivermectin act on nervous system leading to flaccid paralysis and death of mosquitoes [29, 31]. Another possibility could be that ivermectin may inhibit or delay heme detoxification to haematin in the mosquito abdomen that results to small amounts of defecated haematin, and increased heme toxicity that reduce mosquito survival; therefore, further investigations are required to confirm this possibility. Our results are similar to previous studies which found that ivermectin-treated cattle significantly reduced the survival of *Anopheles* mosquitoes by >80% than control cattle in the first week, and the effect gradually declined up until 4 weeks after subcutaneous injection [55–59]. In contrast, Poche et al. [58] reported that oral administration of ivermectin to cattle significantly reduced the survival of *An. arabiensis* than control cattle for up to 1 week post-treatment. Like orally-treated cattle, many previous studies found that orally-treated humans reduced the survival of *Anopheles* mosquitoes for at least 1 week [35, 40, 42, 44, 85].

However, the subcutaneous implants containing ivermectin may extend the negative effects of ivermectin-treated blood on mosquito survivorship for up to 24 weeks after treatment [86]. The long lasting effects of ivermectin on the survival of mosquitoes may be linked with the fact that subcutaneous injection distribute large amount of ivermectin to adipose tissues than oral route, where it slowly released into the peripheral blood circulations which are available to mosquitoes [38–40, 86]. These results suggest that treating cattle with long-lasting subcutaneous of ivermectin may sustain strong reduction in survival of *An. arabiensis*.

Our findings suggest that ivermectin-treated cattle has great potential of controlling residual transmission of malaria by reducing vector density, survival, and vector competence of *An. arabiensis*. The present study revealed that the ivermectin-treated cattle could reduce egg production in *An. arabiensis* for at least 2 weeks. This suggests that mosquitoes would produce fewer eggs after feeding on ivermectin-treated cattle leading to reduced density of adult mosquitoes in the subsequent generations. Additionally, this study also revealed that ivermectin-treated cattle reduced probability of survival and median survival times of *An. arabiensis* for up to 3 weeks. For example, ivermectin treated cattle killed >80% of mosquitoes within 2–4 days post-feeding. These findings indicate that majority of mosquitoes will die before completing egg productions [71, 72], and *Plasmodium falciparum* development to infective sporozoites (10–14 days) [87, 88]. The effects of ivermectin on digestion of blood meal in the stomach of surviving mosquitoes suggests that it may also inhibit establishment of parasite development in mosquitoes [37]. In contrast, the MDA to humans contributed to decreased malaria transmission by reducing survival of wild Anopheles mosquitoes for 1 week that consequently shifted age structure to young females (less infectious mosquitoes), and reduced sporozoite rates for at least 2 weeks [39, 44, 45]. Therefore, the long lasting effects of ivermectin-treated cattle could similarly reduce the vectorial capacity of *An. arabiensis* in the field, and further investigations are required. This study also suggests that treating non-lactating cattle (i.e. calves, heifers and bulls) with long-lasting formulations of ivermectin for large-scale malaria vector control may be the best alternative because it allows milk and meat consumption by the communities in rural settings.

The potential limitation of the experimental design was that the population of *An. arabiensis* within one chamber of the SFS was exposed to untreated or ivermectin-treated cattle in alternating nights, and sample of blood fed mosquitoes were collected in the morning (Table 1). Under this system, some of mosquitoes missed in prior collection may be mixed to those of new night leading to systemic bias (carrying-over effects) in mosquito sample between treatments. Ideally, these experiments were to be conducted in two different chambers (untreated and ivermectin-treated) to avoid mixing samples between treatments, but it was logistically impossible to establish several chambers with *An. arabiensis* populations during dry seasons. Nevertheless, visual observation of mosquito blood meal digestion status in their abdomen was used as a marker to separate between fresh blood-fed mosquitoes from those fed prior nights. The fresh blood fed mosquitoes (i.e. within 12 h) have full reddish/dark red abdomen, but those with two-third black to no blood contents abdomen (semi-gravid and gravid) were considered fed prior nights (i.e. >30 h) [74, 75]. Therefore, these semi-gravid and gravid females were excluded from the sample of fresh blood fed mosquitoes. Besides, our experimental design considered experimental nights, and individual cows as random effects to control for the variations of mosquito catches between nights and individual hosts.

This study confirms that ivermectin-treated cattle reduce blood meal digestion, and subsequent egg production, and survival of *An. arabiensis* for up to 3 weeks in South-eastern Tanzania. These results suggest long lasting effects of ivermectin-treated cattle treated may sustainably suppress *An. arabiensis*, and reduce outdoor transmission of malaria. To ensure continued milk and meat consumption in communities, this study recommends that non-lactating cattle (i.e. calves, heifers and bulls) could be treated with ivermectin for large-scale malaria vector control in villages. Furthermore, the simulation model is under-way to predict the impact of ivermectin-treated cattle alone, or in combination with LLIN/IRS on reducing residual transmission of malaria.
